# Development of mitochondrial DNA cytochrome *c* oxidase subunit I primer sets to construct DNA barcoding library using next-generation sequencing

**DOI:** 10.3897/BDJ.12.e117014

**Published:** 2024-06-18

**Authors:** Seikan Kurata, Shota Mano, Naoyuki Nakahama, Shun K Hirota, Yoshihisa Suyama, Motomi Ito

**Affiliations:** 1 Tomakomai Experimental Forest, Hokkaido University, Tomakomai, Japan Tomakomai Experimental Forest, Hokkaido University Tomakomai Japan; 2 Faculty of Bioresource Sciences, Akita Prefectural University, Akita, Japan Faculty of Bioresource Sciences, Akita Prefectural University Akita Japan; 3 Institute of Natural and Environmental Sciences, University of Hyogo, hyogo, Japan Institute of Natural and Environmental Sciences, University of Hyogo hyogo Japan; 4 Botanical Gardens, Osaka Metropolitan University, Osaka, Japan Botanical Gardens, Osaka Metropolitan University Osaka Japan; 5 Graduate School of Agricultural Science, Tohoku University, Miyagi, Japan Graduate School of Agricultural Science, Tohoku University Miyagi Japan; 6 Komaba museum, University of Tokyo, Tokyo, Japan Komaba museum, University of Tokyo Tokyo Japan

**Keywords:** biodiversity, DNA barcoding, mtDNA COI, insect, next-generation sequencing

## Abstract

Insects are one of the most diverse eukaryotic groups on the planet, with one million or more species present, including those yet undescribed. The DNA barcoding system has been developed, which has aided in the identification of cryptic species and undescribed species. The mitochondrial cytochrome *c* oxidase I region (mtDNA COI) has been utilised for the barcoding analysis of insect taxa. Thereafter, next-generation sequencing (NGS) technology has been developed, allowing for rapid acquisition of massive amounts of sequence data for genetic analyses. Although NGS-based PCR primers designed to amplify the mtDNA COI region have been developed, their target regions were only a part of COI region and/or there were taxonomic bias for PCR amplification. As the mtDNA COI region is a traditional DNA marker for the DNA barcoding system, modified primers for this region would greatly contribute to taxonomic studies. In this study, we redesigned previously developed PCR primer sets that targetted the mtDNA COI barcoding region to improve amplification efficiency and to enable us to conduct sequencing analysis on NGS. As a result, the redesigned primer sets achieved a high success rate (> 85%) for species examined in this study, covering four insect orders (Coleoptera, Lepidoptera, Orthoptera and Odonata). Thus, by combining the primers with developed primer sets for 12S or 16S rRNA regions, we can conduct more detailed taxonomic, phylogeographic and conservation genetic studies using NGS.

## Introduction

Biodiversity can be categorised into three levels: species diversity (richness), genetic diversity and ecosystem diversity. Species richness provides a straightforward method for describing community and regional diversity (Magurran 1988, Gotelli and Colwell 2001). An estimated 10 million species live on Earth (May 1988, May 1990, Stork 1993, Gaston 1991, Costello et al. 2011, Costello et al. 2013), although the exact number is unknown. In recent years, climate change due to anthropogenic effects has increased the risk of extinction for many living species. In addition, many species were likely exterminated before being described, even if the number of living organisms is underestimated. Insects are amongst the most diverse eukaryotic groups on the planet and at least one million species have been described (Stork 2018, Takenaka et al. 2023). Nevertheless, the 1–2% of insect species may be cryptic (Stork 2018) and many new and cryptic species have been described recently (e.g. Takenaka et al. (2023)).

The DNA barcoding system was developed to identify species through DNA sequencing (Hebert et al. 2003, Hebert and Gregory 2005). This innovative system facilitates rapid, accurate, automatable species identification using short standardised gene regions as internal species tags (Hebert and Gregory 2005). DNA barcoding and DNA sequencing approaches have contributed to the detection of cryptic and undescribed species (e.g. Burns et al. (2008), Rebijith et al. (2013)).

Hebert et al. (2003) identified the mitochondrial cytochrome *c* oxidase I (mtDNA COI) region as the core target region for DNA barcoding because it robustly detects moderate genetic differences amongst species as a marker in taxonomic and phylogenetic studies. The 12S and 16S ribosomal RNA loci are also used to identify specimens (e.g. Marquina et al. (2018), Takenaka et al. (2023)). Using genetic differences in the COI region, phylogeographic and conservation genetic studies of many insect species have been conducted (e.g. Buckley et al. (2009), Nakahama et al. (2018), Çıplak et al. (2022)), allowing access to substantial reference sequences in the International Nucleotide Sequence Database Collaboration (INSDC), which involves the DNA Data Bank of Japan (DDBJ), European Molecular Biology Laboratory (EMBL) and the National Center for Biotechnology Information (NCBI). The COI region is more suitable for DNA barcoding than other mtDNA loci, specifically the 658-bp sequence amplified by the primer pair LCO1490 and HCO2198 established by Folmer et al. (1994), which has been widely used for various insect taxa. With the development of next-generation sequencing (NGS), it has become possible to obtain large numbers of short sequence reads (ca. 300 bp) rapidly. Therefore, the modification of primer sets to amplify the COI region efficiently across a diverse range of insect taxa and to conduct NGS sequencing would enhance reference sequences in the region for DNA barcoding.

Although primer sets have been developed to amplify the COI region for NGS-based analysis (i.e. DNA metabarcoding; for example, Leray et al. (2013); Meier et al. (2015); Elbrecht and Leese (2017)), contributing to the identification of insect species, these primer sets are not suitable for enhancing reference sequences in the COI region because they amplify only part of the COI region. Furthermore, insect species have not been identified for which these primer sets are suitable. Although Zhou et al. (2013), Liu et al. (2017) and Yang et al. (2020) developed new NGS-based pipelines of the mtDNA COI region, the specimens sampled in each analysis showed taxonomic bias, i.e. specimens were selected only from Lepidoptera, Diptera and Neuroptera, respectively. Therefore, there is a need to develop primer sets for NGS-based analysis that can amplify the full length of the COI barcoding region and are applicable to many insect taxa. Novel primer sets for NGS-based analysis that amplify the 12S and 16S ribosomal RNA loci have been developed and used to detect cryptic species in some insect taxa, including 11 orders, 42 families and 70 species (Takenaka et al. 2023). More recently, multiplexed phylogenetic marker sequencing (MPM-seq) was developed (Suyama et al. 2021), to enable the simultaneous detection multi-locus sequences. Thus, the development of primer sets targetting insect mtDNA could contribute to research on taxonomy, phylogeography and conservation genetics. In this study, we redesigned previously developed primer sets that targeted the entire COI barcoding region, which will enhance COI reference sequences for insect taxa.

## Materials and Methods

### Development of primer sets

**Figure 1.**
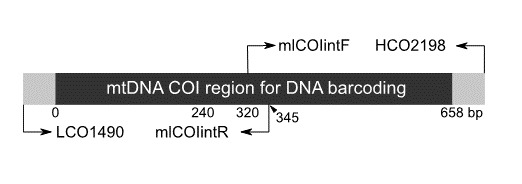
Imaged position of the primers in this study.

To identify polymorphic sites in the primer annealing regions of different insect taxa, we downloaded COI sequences from the NCBI database (Suppl. material 1; 33 species, 29 families and 14 orders). These 33 sequences were aligned using MAFFT v.7.310-1 (Katoh 2002, Katoh and Standley 2013). Polymorphic sites within the COI region across the 33 species were visualised using MEGA-X (Kumar et al. 2018), which provided information for the modification of the primer pair to include mixed bases (e.g. A/G: R, A/T/C:H). Given that the total sequence amplified by this primer pair exceeds 500 bp, the resultant sequence reads are unsuitable for NGS-based sequence analysis. To address this problem, we integrated an intermediate primer pair, mlCOIintF and mlCOIintR (Leray et al. 2013), to distinguish the first half of the COI region in this study (1-319 bp, Fig. 1, Table 1). An approximately 240-bp portion of the COI region, which is moderately conserved amongst species (Leray et al. 2013), was selected as the annealing site for the forward primer of the second half of the COI region (262–658 bp, Fig. 1, Table 1). The sites were also modified as described above, giving rise to two primer pairs; thus, modified LCO1490 and mlCOIintR amplify the first half of the COI region and the new forward primer COmfd_F and modified HCO2198 amplify the second half of the COI region (Table 1), with expected amplification products of approximately 350 bp.

### Sample collection

Between April to October 2022, we collected 96 specimens comprising 96 species, 48 families and 11 orders (Table 2). Each specimen was preserved by freezing.

### PCR amplification and sequencing analysis

Genomic DNA was extracted using DNeasy Blood & Tissue Kits (QIAGEN, Hilden, Germany) and the total DNA concentration was quantified with a NanoDrop ND-1000 (Thermo Scientific, Waltham, USA). Polymerase chain reaction (PCR) was performed for each specimen according to the manufacturer’s protocol, in a final volume of 10 µl that included 5–10 ng of DNA, 1.0 µl Ex Taq Buffer, 0.2 µmol/l of primers, 0.8 µl of dNTP mixture (2.5 mM of each dNTP), 2 U of Takara Ex Taq polymerase (Takara Bio, Otsu, Japan) and sterile distilled water up to 10 µl. The PCR thermal cycling conditions were an initial 1 min denaturation at 94°C; 35 cycles of 94°C for 30 s, 52°C for 30 s, 72°C for 1 min, with a final 20-min extension at 72°C. The PCR product was verified using a MultiNA microchip electrophoresis system (SHIMADZU, Kyoto, Japan). Before sequencing, the PCR products (i.e. LCO1490–COmfd_R and COmfd_F–HCO2198) were pooled for each specimen sample and all 96 samples were prepared for sequencing. Subsequent paired-end sequencing was conducted using 2 × 250 bp cycle run on an Illumina MiSeq Sequencer (Illumina, San Diego, USA) and with the MiSeq Reagent Nano Kit v.2 (500 cycles).

### Assembling sequences and phylogenetic analysis

Data preprocessing, quality control and identification of representative sequences were conducted using Claident v.0.2.2019.05.10 (Tanabe and Toju 2013), as described by Suyama et al. (2021).

Before quality control and data analysis using Claident, non-demultiplexed fastq files (261 bp) were produced from BCL files using *bcl2fastq* v.1.8.4 (Illumina). During this step, non-demultiplexed fastq reads were sorted, based on index reads (index1: 9 bp, index2: 5 bp) and the last position of the raw reads was trimmed (--use-bases-mask Y260n,I9,I5,Y260n). Subsequently, the non-multiplexed fastq reads were demultiplexed using the Claident command *clsplitseq*, which specifies indices and primer sequences and the quality threshold of the index sequence was set to 30 (--minqualtag=30). As the modified primers included 0–3 Ns for accommodation, the option (--truncateN=enable) was used. Files output from *clsplitseq* were deposited in the DDBJ (accession no. DRA017438).

As paired-end sequencing was used, it was possible to identify overlaps between forward and reverse reads. The *clconcatpair* command with the --mode=OVL argument was used to generate concatenated reads from the forward and reverse sequences. Any low-quality reads were filtered out using the *clfilterseq* command with settings --maxplowequal=0.1 --minqual=27, to remove positions with quality lower than Q27.

Further cleaning to remove noisy and chimeric sequences was performed using the following *clcleanseqv* parameters: --derepmode=FULLLENGTH --primarymaxnmismatch=0 --secondarymaxnmismatch=1 --pnoisycluster=0.5. Representative sequences for each sample were identified using the *clclasseqv* command with a 99% identity threshold (--minident=0.99). However, the sequences output by *clclasseqv* remained separated into two regions.

Overlap was detected and the sequences from the two loci were merged using the EMBOSS programme (Rice et al. 2000). The merged sequences were aligned using MAFFT v.7.310-1, and phylogenetic analysis was conducted using the Maximum-Likelihood method in IQ-TREE (Nguyen et al. 2014). The best substitution model was selected using the ModelFinder Plus option (-m MFP) and the GTR+F+I+G4 model was identified as giving the best fit according to the Bayesian Information Criterion (BIC). Additionally, the ultrafast bootstrap approximation and Shimodaira–Hasegawa approximate likelihood ratio test (SH-aLRT) were set to 1000 replicates to assess branch reliability (-bb 1000 and -alrt 1000). Three Collembola sequences (accession nos.: JN970939.1, MF916630.1 and KY829298.1) were included as outgroup. Phylogenetic analysis using the neighbour-joining method (Saitou and Nei 1987) was also conducted with MEGA-X under the substitution model of the Jukes–Cantor model. The consensus trees were visualised and edited using FigTree v.1.4.3 (Rambaut 2016). A BLAST search was also conducted using reference sequences in GenBank.

## Results and Discussion

We redesigned two primer sets (LCO1490–COmfd_R and COmfd_F–HCO2198) that amplified the DNA barcoding region of the mtDNA COI region of insect taxa (see Table 1). The original barcoding primers for the mtDNA COI region are unsuitable for NGS-based analysis due to their excessive sequence length. Therefore, we modified the original primers (Folmer et al. 1994) and designed internal primers such as mlCOIintF/R (Leray et al. 2013) to amplify lengths appropriate for NGS-based analysis. The sequence reads were divided into two parts: a 319-bp fragment from the PCR amplification products of LCO1490–COmfd_R and a 397-bp fragment from COmfd_F–HCO2198. Our PCR amplification trial and sequence analysis indicated that our modified primer sets successfully amplified the mtDNA COI region, demonstrating the effectiveness of these primer sets for taxonomic, phylogeographic and conservation genetic studies of insect taxa.

### Universality and efficiency of the modified primer sets

Using the modified COI primer sets for DNA barcoding, we conducted PCR to amplify samples from 96 species, encompassing 48 families and 11 orders and performed NGS sequencing analysis. These primers successfully amplified and sequenced the target mtDNA COI regions of 80 species from 41 families in 11 orders. Notably, the primer sets had high success rates for Coleoptera, Lepidoptera, Orthoptera and Odonata (Table 3). Despite moderate sequencing success rates and limited specimen samples, the primer sets also showed promise as effective barcoding primers for Hymenoptera, Hemiptera and Diptera.

After the clustering step in Claident, the modified barcoding primers generated 12–18024 (median, 1102; average, 2774) and 2–18002 (median, 1060; average, 2720) reads using LCO1490–COmfd_R and COmfd_F–HCO2198, respectively (Table 2, Suppl. material 2). There was a pronounced bias in the number of reads between LCO1490–COmfd_R and COmfd_F–HCO2198 (Table 2). However, the high degree of variability in the mtDNA COI region (Leray et al. 2013) suggests that this bias may be due to variability at the primer annealing sites.

**Figure 2.**
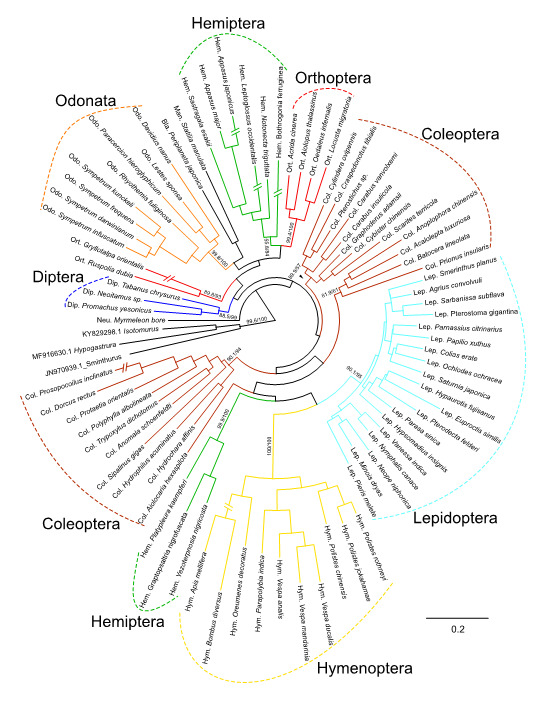
The Maximum-Likelihood (ML) phylogenetic tree, based of mtDNA COI region which originated from sequence reads of the modified primer sets. The numbers on the major branches represents bootstrap values from the ultrafast bootstrap replications and SH-aLRT methods, respectively. The horizontal scale bar under the tree represents evolutionary distance between specimen taxa. Headers of scientific names represent abbreviations of the order names (e.g. Col. and Hym.).

To assess whether the modified barcoding primer sets could differentiate various insect taxa, we conducted phylogenetic analysis. The resulting phylogenetic tree showed that related insect taxa clustered within the same lineages (Fig. 2, Suppl. material 3). However, three orders were paraphyletic: Coleoptera, Hemiptera and Orthoptera (Fig. 2). The phylogenetic relationships amongst orders cannot be fully elucidated using a single locus, particularly when only short fragment sequences are available. Takenaka et al. (2023) also reported paraphyly of Coleoptera and Hemiptera. Therefore, we conclude that these phylogenetic results are not major issues within the scope of this study. Nevertheless, our results suggest that we obtained accurate sequences, as related species were identified as candidate sequences in BLAST searches (Suppl. material 4) and the primer sets appear to be suitable for insect barcoding analyses. We also directly compared additional Chironomid NGS assemblies whose DNA template libraries were similar to the sequence data from the Chironomid DNA Barcode Database (https://www.nies.go.jp/yusurika/en/contents/search.php). Although we performed NGS sequencing analysis of 16 Chironomid samples, we obtained complete mtDNA COI assemblies from 13 Chironomid specimen samples (Suppl. material 5). Comparing these 13 Chironomid NGS assemblies against the database, we detected no assembly errors (Suppl. material 5).

### Future utilisation of the modified COI primer sets

The goal of our research was to modify the existing mtDNA COI primer set established by Folmer et al. (1994) for use in NGS sequencing analysis to enhance DNA barcoding reference databases. The original primer set of Folmer et al. (1994) is foundational to the DNA barcoding system and has been widely used in taxonomic, phylogeographic and conservation genetic studies. Recently, Leese et al. (2020) developed new primer sets for the mtDNA COI region tailored for NGS-based analysis. However, the sequences obtained with these new primers were shorter than those produced by the original primer set, leading to concerns that they might not be as effective for enhancing DNA barcoding references. In this study, we modified the original primer set of Folmer et al. (1994). The modified primer sets are anticipated to greatly enhance DNA barcoding references, although these primers are not compatible with some insect taxa, as indicated in Table 2. Shokralla et al. (2015) also developed NGS-based universal primer sets for the mtDNA COI region; however, their success rate was ca. 70% for all insect taxa. Although the current study examined somewhat limited specimen samples, the success rate was 80% (Table 3). Notably, the success rate for Coleoptera was significantly higher than that reported by Shokralla et al. (2015), whereas our success rate was similar to that of Liu et al. (2013). However, because the primer sequences differ, the modified primer sets should be useful as supplemental primer sets for those of Liu et al. (2013) and Shokralla et al. (2015).

Advances in NGS system have led to various NGS applications in ecological studies. Suyama et al. (2021) introduced the multiplexed phylogenetic marker sequencing (MPM-seq) technique, which enables the simultaneous acquisition of genetic information using multiple primer sets. Takenaka et al. (2023) developed innovative primer sets targetting the mtDNA 16S and 12S rRNA regions, contributing to the discovery of cryptic species and previously undescribed species. As these primer sets generate short sequences, they are also suited for NGS-based analysis (Takenaka et al. 2023). Thus, using our modified primer sets for mtDNA COI, 16S rRNA and 12S rRNA in conjunction with MPM-seq allows more comprehensive taxonomic, phylogeographic and conservation genetic studies.

The mtDNA COI region is considered challenging for designing new NGS-based primer sets due to the high polymorphism rate (Deagle et al. 2014, Takenaka et al. 2023). This complexity has led to low sequencing success rates for insect taxa such as Hymenoptera and Hemiptera (Table 3). In this study, we designed primers manually by visualising primer annealing sites for 33 insect taxa (Suppl. material 1), without performing *in silico* analysis. We anticipate that further modified primer sequences will enhance the success rates of PCR amplification and sequencing analysis. Nevertheless, due to the presence of mixed bases in the primer sequences, which may lead to the amplification of non-target loci, the primer sequences must be redesigned with caution.

## Supplementary Material

5D8E5DD8-FCB1-5B10-A995-2CA515F92745 10.3897/BDJ.12.e117014.suppl1 Supplementary material 1 Sequence information for designing PCR primers Data type Table Brief description We designed the PCR primers by aligning the sequences. File: oo_943449.docx https://binary.pensoft.net/file/943449 Seikan Kurata, Shota Mano, Naoyuki Nakahama, Shun K. Hirota, Yoshihisa Suyama, Motomi Ito

3BE148F7-ABC9-5C3A-A94F-CFF6B7356608 10.3897/BDJ.12.e117014.suppl2 Supplementary material 2 The number of reads after quality control or data analyses using Claident Data type Table File: oo_943450.docx https://binary.pensoft.net/file/943450 Seikan Kurata, Shota Mano, Naoyuki Nakahama, Shun K. Hirota, Yoshihisa Suyama, Motomi Ito

AF8ADC28-C96D-59F6-8589-A0AD982FC3E7 10.3897/BDJ.12.e117014.suppl3 Supplementary material 3 Phylogenetic tree using the neighbour-joining method Data type Figure Brief description The neighbour-joining (NJ) tree based of mtDNA COI region which originated from sequence reads of the modified primer sets. File: oo_1061207.docx https://binary.pensoft.net/file/1061207 Seikan Kurata, Shota Mano, Naoyuki Nakahama, Shun K. Hirota, Yoshihisa Suyama, Motomi Ito

8B01C3A8-B0B6-5A22-8F0A-0CC815374E4A 10.3897/BDJ.12.e117014.suppl4 Supplementary material 4 The number of reads from first half (LCO1490–COmfd_R: 1-319) and second half (COmfd_F–HCO2198: 262-658) of the COI region, and the results of BLAST search. Data type Table Brief description The number of reads from first half (LCO1490–COmfd_R: 1-319) and second half (COmfd_F–HCO2198: 262-658) of the COI region. There is a great difference for the number of reads between the most dominant and the second largest read. In addition, the results of the BLAST search were shown on the right side in the Table. File: oo_994778.docx https://binary.pensoft.net/file/994778 Seikan Kurata, Shota Mano, Naoyuki Nakahama, Shun K. Hirota, Yoshihisa Suyama, Motomi Ito

D8C3EC5F-3C06-529C-A05B-6C56AB1F6E11 10.3897/BDJ.12.e117014.suppl5 Supplementary material 5 Comparison results of Chironomid NGS assemblies against the sequence data from the Chironomid DNA Barcode Database by the National Institute for Environmental Studies (NIES). Data type Table Brief description Comparison results of Chironomid NGS assemblies against the sequence data from Sanger sequencing. File: oo_1049788.docx https://binary.pensoft.net/file/1049788 Seikan Kurata, Shota Mano, Naoyuki Nakahama, Shun K. Hirota, Yoshihisa Suyama, Motomi Ito

## Figures and Tables

**Table 1.** Information of newly-modified primer sets in this study.

Primer name	Primer sequence (5’ –3’)*^1^	Target region
LCO1490	TCWACWAAYCAYAARGAYATYGG	1–319 bp of the mtDNA COI
COmfd_R	GGDGGRTANAHHGTTCAHCCNGTHCC	
COmfd_F	CCNCGRHTRAAYAAYATRAGWTTYTG	262–658 bp of the mtDNA COI
HCO2198	ACTTCDGGRTGNCCAAARAAYCA	

**Table 2.** Specimen samples and the result of sequencing analysis.

Specimen samples information			Number of reads*^2^		Sampling location		
Order	Family	species	1-319 bp	262-658 bp	Locality name	Latitude	Longitude
Coleoptera	Carabidae	*Carabusvanvolxemi* Putzeys, 1875	2839	3520	Japan: Mt. Moriyoshi	39.99°N	140.60°E
	Carabidae	*Carabusinsulicola* Chaudoir, 1869	4035	2954	Japan: Akita, Yamamoto, Mitane	40.06°N	140.11°E
	Carabidae	*Craspedonotustibialis* Schaum, 1863	149	102	Japan: Akita, Akita, Shimoshinjo	39.80°N	140.05°E
	Carabidae	*Cylinderaovipennis* Bates, 1883	131	215	Japan: Akita, Akita, Nibetsu	39.80°N	140.22°E
	Carabidae	*Scaritesterricola* Bonelli, 1813	6141	2384	Japan: Akita, Minamiakita, Ogata	40.01°N	139.95°E
	Carabidae	*Pterostichus* Bonelli, 1810	3746	1547	Japan: Akita, Akita, Shimoshinjo	39.80°N	140.05°E
	Ceranbycidae	*Acaloleptaluxuriosa* Bates, 1873	130	24	Japan: Akita, Minamiakita, Ogata	40.01°N	139.95°E
	Ceranbycidae	*Anoplophorachinensis* Forster, 1771	18024	15712	Japan: Akita, Akita, Nibetsu	39.80°N	140.22°E
	Ceranbycidae	*Batoceralineolata* Chevrolat, 1852	7707	2946	Japan: Akita, Minamiakita, Ogata	40.01°N	139.95°E
	Ceranbycidae	*Prionusinsularis* Motschulsky, 1857	980	610	Japan: Akita, Minamiakita, Ogata	40.01°N	139.95°E
	Coccinellidae	*Aiolocariahexaspilota* Hope, 1831	23	24	Japan: Aktia, Akita, Toyoiwatoyomaki	39.64°N	140.11°E
	Dytiscidae	*Cybisterchinensis* Motschulsky, 1854	109	2	Japan: Akita, Noshiro, Asanai	40.15°N	140.04°E
	Dytiscidae	*Graphoderusadamsii* Clark, 1864	57	9	Japan: Akita, Noshiro, Asanai	40.15°N	140.04°E
	Dytiscidae	*Rhantussuturalis* MacLeay, 1825	－	－	Japan: Akita, Akita, Shimoshinjo	39.80°N	140.05°E
	Hydrophilidae	*Hydrocharaaffinis* Sharp, 1873	8020	7971	Japan: Akita, Minamiakita, Ogata	40.01°N	139.95°E
	Hydrophilidae	*Hydrophilusacuminatus* Motschulsky, 1854	8335	11208	Japan: Akita, Minamiakita, Ogata	40.01°N	139.96°E
	Lucanidae	*Dorcusrectus* Motschulsky, 1858	326	835	Japan: Akita, Minamiakita, Ogata	40.01°N	139.95°E
	Lucanidae	*Prosopocoilusinclinatus* Motschulsky, 1857	1184	2856	Japan: Akita, Minamiakita, Ogata	40.01°N	139.95°E
	Rhynchophoridae	*Sipalinusgigas* Fabricius, 1775	2783	1162	Japan: Akita, Minamiakita, Ogata	40.01°N	139.95°E
	Scarabaeidae	*Anomalaschoenfeldti* Ohaus, 1915	536	337	Japan: Akita, Akita, Shimoshinjo	39.80°N	140.05°E
	Scarabaeidae	*Polyphyllaalbolineata* Motschulsky, 1861	1525	3223	Japan: Akita, Minamiakita, Ogata	40.01°N	139.95°E
	Scarabaeidae	*Protaetiaorientalis* Gory & Percheron, 1833	5218	7267	Japan: Akita, Akita, Shimoshinjo	39.80°N	140.05°E
	Scarabaeidae	*Trypoxylusdichotomus* Linnaeus, 1771	1673	1417	Japan: Akita, Minamiakita, Ogata	40.01°N	139.95°E
	Tenebrionidae	*Cryphaeusamurensis* Heyden, 1884	－	－	Japan: Akita, Yamamoto, Mitane	40.06°N	140.11°E
Hymenoptera	Apidae	*Apismellifera* Linnaeus, 1758	66	1727	Japan: Akita, Akita, Shimoshinjo	39.80°N	140.05°E
	Apidae	*Bombusdiversus* Smith, 1869	608	727	Japan: Aomori, Shimokita, Higashidori	41.37°N	141.44°E
	Apidae	*Bombusterrestris* Linnaeus, 1758	－	－	Japan: Hokkaido, Hakodate, Bandai	41.79°N	140.73°E
	Pompilidae	*Auplopuscarbonarius* Scopoli, 1763	－	－	Japan: Mt. Moriyoshi	39.99°N	140.60°E
	Scoliidae	*Scoliahistrionica* Smith, 1873	－	－	Japan: Aomori, Shimokita, Higashidori	41.37°N	141.44°E
	Vespidae	*Oreumenesdecoratus* Smith, 1852	443	268	Japan: Yamagata, Tsuruoka, Obari	38.56°N	139.86°E
	Vespidae	*Parapolybiaindica* Saussure, 1854	1612	1106	Japan: Akita, Akita, Kanaashi	39.81°N	140.07°E
	Vespidae	*Polisteschinensisantennalis* Yamane, 1972	26	9	Japan: Akita, Akita, Shimoshinjo	39.80°N	140.05°E
	Vespidae	*Polistesjokahamae* Radoszkowski, 1887	3867	3411	Japan: Akita, Akita, Shimoshinjo	39.80°N	140.05°E
	Vespidae	*Polistesrothneyi* Cameron, 1900	626	338	Japan: Akita, Akita, Shimoshinjo	39.80°N	140.05°E
	Vespidae	*Vespaanalis* Fabricius, 1775	1090	868	Japan: Akita, Minamiakita, Ogata	40.01°N	139.95°E
	Vespidae	*Vespaducalis* Smith, 1852	1572	1669	Japan: Yamagata, Shinjo, Tsunozawa	38.71°N	140.26°E
	Vespidae	*Vespamandarinia* Smith, 1852	7871	12385	Japan: Akita, Akita, Shimoshinjo	39.80°N	140.05°E
Lepidoptera	Agaristidae	*Sarbanissasubflava* Moore, 1877	4388	2931	Japan: Akita, Minamiakita, Ogata	40.01°N	139.95°E
	Callidulidae	*Pterodectafelderi* Bremer, 1864	3011	8868	Japan: Yamagata, Tsuruoka, Obari	38.56°N	139.86°E
	Drepanidae	*Hypsomadiusinsignis* Butler, 1877	858	1338	Japan: Akita, Minamiakita, Ogata	40.01°N	139.95°E
	Heterogeneidae	*Parasasinica* Moore, 1877	3105	1538	Japan: Akita, Minamiakita, Ogata	40.01°N	139.95°E
	Lycaenidae	*Lycaenaphlaeas* Linnaeus, 1761	48	－	Japan: Akita, Akita, Shimoshinjo	39.80°N	140.05°E
	Lycaenidae	*Hypaurotisfujisanus* Matsumura, 1910	1124	307	Japan: Yamagata, Tsuruoka, Obari	38.56°N	139.86°E
	Lymantriidae	*Euproctissimilis* Fuessly, 1775	36	19	Japan: Akita, Minamiakita, Ogata	40.01°N	139.95°E
	Notodontidae	*Pterostomagigantina* Staudinger, 1892	1110	65	Japan: Akita, Minamiakita, Ogata	40.01°N	139.95°E
	Nymphalidae	*Nymphaliscanace* Linnaeus, 1763	2009	1002	Japan: Aichi, Tahara, Nishiyama	34.60°N	137.05°E
	Nymphalidae	*Minoisdryas* Scopoli, 1763	1000	503	Japan: Aomori, Kitatsugaru, Nakadomari	41.19°N	140.34°E
	Nymphalidae	*Neopeniphonica* Butler, 1881	10303	8532	Japan: Mt. Moriyoshi	39.99°N	140.60°E
	Nymphalidae	*Ochlodesochracea* Bremer, 1861	1295	2339	Japan: Iwate, Kunohe, Kunohe	40.14°N	141.39°E
	Nymphalidae	*Vanessaindica* Herbst, 1794	12816	5480	Japan: Yamagata, Tsuruoka, Wasada	38.57°N	139.56°E
	Papilionidae	*Papilioxuthus* Linnaeus, 1767	1019	1520	Japan: Aichi, Tahara, Nishiyama	34.60°N	137.05°E
	Papilionidae	*Parnassiuscitrinarius* Butler, 1866	8379	11203	Japan: Yamagata, Tsuruoka, Wasada	38.57°N	139.56°E
	Pieridae	*Coliaserate* Esper, 1805	6181	1115	Japan: Akita, Yurihonjo, Tsuchiya	39.39°N	140.07°E
	Pieridae	*Pierismelete* Menetries, 1857	4541	6119	Japan: Iwate, Kunohe, Kunohe	40.14°N	141.39°E
	Saturniidae	*Saturniajaponica* Moore, 1872	4500	8871	Japan: Akita, Minamiakita, Ogata	40.01°N	139.95°E
	Sphindidae	*Agriusconvolvuli* Linnaeus, 1758	5921	10323	Japan: Akita, Akita, Shimoshinjo	39.80°N	140.05°E
	Sphindidae	*Smerinthusplanus* Walker, 1856	4596	703	Japan: Akita, Minamiakita, Ogata	40.01°N	139.95°E
Hemiptera	Acanthosomatidae	*Sastragalaesakii* Hasegawa, 1959	137	33	Japan: Akita, Akita, Nibetsu	39.80°N	140.22°E
	Belostomatidae	*Appasusjaponicus* Vuillefroy, 1864	201	641	Japan: Aktia, Akita, Toyoiwatoyomaki	39.64°N	140.11°E
	Belostomatidae	*Appasusmajor* Esaki, 1934	88	93	Japan: Aktia, Akita, Toyoiwatoyomaki	39.64°N	140.11°E
	Cicadellidae	*Bothrogoniaferruginea* Fabricius, 1787	150	26	Japan: Yamagata, Tsuruoka, Wasada	38.57°N	139.56°E
	Cicadidae	*Graptopsaltrianigrofuscata* Motschulsky, 1866	6556	7179	Japan: Akita, Minamiakita, Ogata	40.01°N	139.95°E
	Cicadidae	*Platypleurakaempferi* Fabricius, 1794	3304	3833	Japan: Akita, Minamiakita, Ogata	40.01°N	139.95°E
	Cicadidae	*Yezoterpnosianigricosta* Motschulsky, 1866	17050	18002	Japan: Mt. Moriyoshi	39.99°N	140.60°E
	Coreidae	*Leptoglossusoccidentalis* Heidemann, 1910	404	74	Japan: Akita, Akita, Shimoshinjo	39.80°N	140.05°E
	Corixidae	*Hesperocorixahokkensis* Matsumura, 1905	－	－	Japan: Akita, Minamiakita, Ogata	40.01°N	139.95°E
	Gerridae	*Aquariuspaludum* Fabricius, 1794	－	－	Japan: Akita, Akita, Shimoshinjo	39.80°N	140.05°E
	Notonectidae	*Notonectatriguttata* Motschulsky, 1861	621	416	Japan: Akita, Akita, Shimoshinjo	39.80°N	140.05°E
	Pentatomidae	*Palomenaangulosa* Motschulsky, 1861	－	－	Japan: Akita, Akita, Shimoshinjo	39.80°N	140.05°E
	Pentatomidae	*Pentatomajaponica* Distant, 1882	－	8	Japan: Iwate, Kunohe, Kunohe	40.14°N	141.39°E
	Reduviidae	*Agriosphodrusdohrni* Stal, 1862	－	－	Japan: Akita, Akita, Shimoshinjo	39.80°N	140.05°E
	Reduviidae	*Ectrychotesandreae* Thunberg, 1784	65	－	Japan: Akita, Akita, Shimoshinjo	39.80°N	140.05°E
	Reduviidae	*Velinusnodipes* Uhler, 1860	－	－	Japan: Akita, Akita, Shimoshinjo	39.80°N	140.05°E
Orthoptera	Acrididae	*Acridacinerea* Thunberg, 1815	5446	9947	Japan: Akita, Akita, Shimoshinjo	39.80°N	140.05°E
	Acrididae	*Aiolopusthalassinus* Fabricius, 1781	13476	7387	Japan: Akita, Akita, Shimoshinjo	39.80°N	140.05°E
	Acrididae	*Locustamigratoria* Linnaeus, 1758	2739	452	Japan: Aichi, Tahara, Nishiyama	34.60°N	137.05°E
	Acrididae	*Oedaleusinfernalis* Saussure, 1884	1095	441	Japan: Akita, Akita, Shimoshinjo	39.80°N	140.05°E
	Gryllotalpidae	*Gryllotalpaorientalis* Burmeister, 1838	2842	3479	Japan: Akita, Minamiakita, Ogata	40.01°N	139.95°E
	Tettigoniidae	*Ruspoliadubia* Redtenbacher, 1891	271	317	Japan: Akita, Minamiakita, Ogata	40.01°N	139.96°E
Diptera	Asilidae	*Neoitamusangusticornis* Loew, 1858	－	－	Japan: Akita, Akita, Shimoshinjo	39.80°N	140.05°E
	Asilidae	*Neoitamus* Osten Sacken, 1878	16	5	Japan: Mt. Moriyoshi	39.99°N	140.60°E
	Asilidae	*Promachusyesonicus* Bigot, 1887	3003	2058	Japan: Akita, Akita, Shimoshinjo	39.80°N	140.05°E
	Tabanidae	*Tabanuschrysurus* Loew, 1858	144	79	Japan: Iwate, Morioka, Yabukawa	39.85°N	141.45°E
Odonata	Coenagrionidae	*Paracercionhieroglyphicum* Brauer, 1865	871	928	Japan: Akita, Katagami, Ten-nou	39.91°N	140.02°E
	Gomphidae	*Davidiusnanus* Selys, 1869	1009	1060	Japan: Iwate, Kunohe, Kunohe	40.14°N	141.39°E
	Lestidae	*Lestessponsa* Hansemann, 1823	31	3	Japan: Iwate, Morioka, Yabukawa	39.85°N	141.45°E
	Libellulidae	*Rhyothemisfuliginosa* Selys, 1883	2078	2880	Japan: Akita, Minamiakita, Ogata	40.01°N	139.95°E
	Libellulidae	*Sympetrumdarwinianum* Selys, 1883	87	107	Japan: Akita, Minamiakita, Ogata	40.01°N	139.95°E
	Libellulidae	*Sympetrumfrequens* Selys, 1883	999	1698	Japan: Akita, Akita, Sotoasahikawa	39.75°N	140.10°E
	Libellulidae	*Sympetruminfuscatum* Selys, 1883	268	712	Japan: Akita, Akita, Shimoshinjo	39.80°N	140.05°E
	Libellulidae	*Sympetrumkunckeli* Selys, 1884	449	1340	Japan: Akita, Akita, Shimoshinjo	39.80°N	140.05°E
Dermaptera	Labiduridae	*Labidurariparia* Pallas, 1773	－	39	Japan: Akita, Akita, Shimoshinjo	39.80°N	140.05°E
Neuroptera	Myrmeleontidae	*Myrmeleonbore* Tjeder, 1941	160	41	Japan: Akita, Minamiakita, Ogata	40.01°N	139.95°E
Mantodea	Mantidae	*Statiliamaculata* Thunberg, 1784	1328	156	Japan: Akita, Minamiakita, Ogata	40.01°N	139.95°E
	Mantidae	*Tenoderaaridifolia* Stoll, 1813	46	－	Japan: Akita, Akita, Shimoshinjo	39.80°N	140.05°E
Blattaria	Blattidae	*Periplanetajaponica* Karny, 1908	395	631	Japan: Akita, Akita, Shimoshinjo	39.80°N	140.05°E

**Table 3.** Success rate of sequence analysis.

Order	No. of specimen samples*^3^	No. of samples*^4^	Success rate (%)
Coleoptera	24	22	91.7
Hymenoptera	14	10	71.4
Lepidoptera	20	19	95
Hemiptera	16	9	56.3
Orthoptera	6	6	100
Diptera	4	3	75
Odonata	9	8	88.9
Others	5	3	–
Total	96	80	82.3

## Footnotes

*1 Illumina adapter sequences (F: CGCTCTTCCGATCTCTG; R: TGCTCTTCCGATCTGAC) were added to the 5’ end of the primer sequence and 0–3Ns were inserted between adapter sequences and primer sequences.

*2 The number of reads from first half (LCO1490–COmfd_R: 1-319) and second half (COmfd_F–HCO2198: 262-658) of the COI region, which were clustered and counted by Claident and the unit is base pair (bp). The symbol of “–” indicates that suitable fragment reads for the mtDNA COI region were not detected.

*3 The number of specimen samples for sequencing analysis.

*4 The number of samples which pass the PCR trials and NGS-based sequencing analysis.
