# Supplementary material for: Development of mitochondrial DNA cytochrome c oxidase subunit I primer sets to construct DNA barcoding library using next-generation sequencing
Source: Biodivers Data J. 2024 Jun 18;12:e117014. doi: 10.3897/BDJ.12.e117014 (PMC11199957; doi:10.3897/BDJ.12.e117014)
Supplement: Supplementary material 1 — Sequence information for designing PCR primers [file bdj-12-e117014-s001.docx]

**Table S1** Sequence information for designing PCR primers.

| Family | Genus | Scientific epithet | Accession No. |
| --- | --- | --- | --- |
| Acrididae | Locusta | migratoria | JN858213.1 |
| Pthiridae | Pediculus | humanus | KC685850.1 |
| Curculionidae | Anthonomus | eugenii | MK654653.1 |
| Vespidae | Allorhynchium | chinense | MT178402.1 |
| Thripidae | Thrips | imaginis | NC_004371.1 |
| Culicidae | Aedes | albopictus | NC_006817.1 |
| Mantidae | Tamolanica | tamolana | NC_007702.1 |
| Syrphidae | Simosyrphus | grandicornis | NC_008754.1 |
| Pentatomidae | Nezara | viridula | NC_011755.1 |
| Ichneumonidae | Diadegma | semiclausum | NC_012708.1 |
| Saturniidae | Eriogyna | pyretorum | NC_012727.1 |
| Formicidae | Solenopsis | richteri | NC_014677.1 |
| Pieridae | Pieris | rapae | NC_015895.1 |
| Chironomidae | Chironomus | tepperi | NC_016167.1 |
| Carabidae | Damaster | mirabilissimus | NC_016469.1 |
| Machilidae | Songmachilis | xinxiangensis | NC_021384.1 |
| Tortricidae | Retinia | pseudotsugaicola | NC_022865.1 |
| Coccinellidae | Henosepilachna | pusillanima | NC_023469.1 |
| Drosophilidae | Drosophila | melanogaster | NC_024511.1 |
| Muscidae | Musca | domestica | NC_024855.1 |
| Capniidae | Apteroperla | tikumana | NC_027698.1 |
| Perlidae | Kamimuria | chungnanshana | NC_028076.1 |
| Staphylinidae | Tetartopeus | terminatus | NC_028613.1 |
| Gelechiidae | Helcystogramma | macroscopa | NC_029844.1 |
| Capniidae | Capnia | zijinshana | NC_034661.1 |
| Formicidae | Anoplolepis | gracilipes | NC_039576.1 |
| Blattidae | Periplaneta | brunnea | NC_039940.1 |
| Entomobryidae | Sinella | curviseta | NC_042755.1 |
| Lucanidae | Dorcus | tenuihirsutus | NC_045124.1 |
| Coccinellidae | Hippodamia | variegata | NC_046481.1 |
| Psychidae | Dahlica | ochrostigma | NC_047459.1 |
| Ephemerellidae | Serratella | zapekinae | NC_050282.1 |
| Ephemerellidae | Torleya | nepalica | NC_050284.1 |
