# Supplementary material for: Development of mitochondrial DNA cytochrome c oxidase subunit I primer sets to construct DNA barcoding library using next-generation sequencing
Source: Biodivers Data J. 2024 Jun 18;12:e117014. doi: 10.3897/BDJ.12.e117014 (PMC11199957; doi:10.3897/BDJ.12.e117014)
Supplement: Supplementary material 2 — The number of reads after quality control or data analyses using Claident [file bdj-12-e117014-s002.docx]

**Table S2** The number of reads after quality control or data analyses using Claident

|  |  | 1-319^*1^ | | 262-658^*1^ | | clfilterseq^*2^ | | clsumclass^*3^ | |
| --- | --- | --- | --- | --- | --- | --- | --- | --- | --- |
| No. |  | F | R | F | R | 1-319 | 262-658 | 1-319 | 262-658 |
| K-01 | *Dorcus rectus* | 2742 | 2742 | 1926 | 1926 | 2717 | 1840 | 326 | 835 |
| K-02 | *Prosopocoilus inclinatus* | 1578 | 1578 | 3950 | 3950 | 1571 | 3891 | 1184 | 2856 |
| K-03 | *Polyphylla albolineata* | 2477 | 2477 | 5317 | 5317 | 2465 | 5145 | 1525 | 3223 |
| K-04 | *Anomala schoenfeldti* | 744 | 744 | 610 | 610 | 742 | 592 | 536 | 337 |
| K-05 | *Cylindera ovipennis* | 194 | 194 | 392 | 392 | 193 | 387 | 131 | 215 |
| K-06 | *Craspedonotus tibialis* | 210 | 210 | 169 | 169 | 210 | 162 | 149 | 102 |
| K-07 | *Carabus vanvolxemi* | 3393 | 3393 | 4786 | 4786 | 3377 | 4729 | 2839 | 3520 |
| K-08 | *Carabus insulicola* | 4928 | 4929 | 4071 | 4071 | 4873 | 4009 | 4035 | 2954 |
| K-09 | *Pterostichus sp.* | 4387 | 4388 | 2409 | 2409 | 4369 | 2338 | 3746 | 1547 |
| K-10 | *Scarites terricola* | 9747 | 9747 | 5883 | 5883 | 9692 | 5734 | 6141 | 2384 |
| K-11 | *Batocera lineolata* | 8979 | 8980 | 5487 | 5487 | 8921 | 5335 | 7707 | 2946 |
| K-12 | *Acalolepta luxuriosa* | 201 | 201 | 63 | 63 | 199 | 60 | 130 | 24 |
| K-13 | *Cybister chinensis* | 158 | 158 | 12 | 12 | 157 | 10 | 109 | 2 |
| K-14 | *Graphoderus adamsii* | 76 | 76 | 24 | 24 | 76 | 14 | 57 | 9 |
| K-15 | *Trypoxylus dichotomus* | 2153 | 2153 | 2173 | 2173 | 2143 | 2136 | 1673 | 1417 |
| K-16 | *Cryphaeus amurensis* | 11 | 11 | 3 | 3 | 11 | 1 | － | － |
| K-17 | *Anoplophora malasiaca* | 20593 | 20597 | 20634 | 20634 | 20459 | 19625 | 18024 | 15712 |
| K-18 | *Hydrophilus acuminatus* | 9948 | 9950 | 14019 | 14019 | 9872 | 13539 | 8335 | 11208 |
| K-19 | *Hydrochara affinis* | 9133 | 9134 | 9845 | 9845 | 9079 | 9591 | 8020 | 7971 |
| K-20 | *Protaetia orientalis* | 6617 | 6619 | 19189 | 19189 | 6584 | 18884 | 5218 | 7267 |
| K-21 | *Rhantus suturalis* | － | － | 8 | 8 | － | － | － | － |
| K-22 | *Sipalinus gigas* | 3610 | 3610 | 1951 | 1951 | 3585 | 1894 | 2783 | 1162 |
| K-23 | *Prionus insularis* | 1261 | 1261 | 993 | 993 | 1255 | 968 | 980 | 610 |
| K-24 | *Aiolocaria hexaspilota* | 34 | 34 | 54 | 54 | 34 | 48 | 23 | 24 |
| K-25 | *Polistes rothneyi* | 821 | 821 | 558 | 558 | 817 | 543 | 626 | 338 |
| K-26 | *Polistes chinensis antennalis* | 42 | 42 | 26 | 26 | 41 | 18 | 26 | 9 |
| K-27 | *Scolia histrionica* | 15 | 15 | 4 | 4 | 15 | － | － | － |
| K-28 | *Bombus diversus* | 835 | 835 | 1161 | 1161 | 828 | 1142 | 608 | 727 |
| K-29 | *Bombus terrestris* | 30 | 30 | 34 | 34 | 30 | 28 | － | － |
| K-30 | *Vespa ducalis* | 1948 | 1948 | 2489 | 2489 | 1939 | 2462 | 1572 | 1669 |
| K-31 | *Oreumenes decoratus* | 630 | 630 | 487 | 487 | 626 | 467 | 443 | 268 |
| K-32 | *Auplopus carbonarius* | 13 | 13 | 14 | 14 | 13 | 9 | － | － |
| K-33 | *Vespa mandarinia* | 8764 | 8764 | 15595 | 15595 | 8687 | 14996 | 7871 | 12385 |
| K-34 | *Vespa analis* | 2503 | 2503 | 2162 | 2162 | 2491 | 2107 | 1090 | 868 |
| K-35 | *Parapolybia indica* | 2075 | 2075 | 1746 | 1746 | 2062 | 1711 | 1612 | 1106 |
| K-36 | *Apis mellifera* | 144 | 144 | 2505 | 2505 | 142 | 2479 | 66 | 1727 |
| K-37 | *Polistes jokahamae* | 4718 | 4718 | 4762 | 4762 | 4681 | 4715 | 3867 | 3411 |
| K-38 | *Smerinthus planus* | 5433 | 5433 | 2332 | 2332 | 5383 | 2296 | 4596 | 703 |
| K-39 | *Parnassius citrinarius* | 9754 | 9755 | 13528 | 13528 | 9644 | 13275 | 8379 | 11203 |
| K-40 | *Neope niphonica* | 11722 | 11728 | 11674 | 11674 | 11614 | 10918 | 10303 | 8532 |
| K-41 | *Vanessa indica* | 14412 | 14413 | 7385 | 7385 | 14300 | 7079 | 12816 | 5480 |
| K-42 | *Minois dryas* | 1341 | 1342 | 875 | 875 | 1319 | 844 | 1000 | 503 |
| K-43 | *Pieris melete* | 5390 | 5390 | 7580 | 7580 | 5355 | 7425 | 4541 | 6119 |
| K-44 | *Ochlodes ochraceus* | 1670 | 1670 | 3286 | 3286 | 1659 | 3233 | 1295 | 2339 |
| K-45 | *Lycaena phlaeas* | 69 | 69 | 4 | 4 | 69 | 3 | 48 | － |
| K-46 | *Sarbanissa subflava* | 5104 | 5104 | 3891 | 3891 | 5068 | 3840 | 4388 | 2931 |
| K-47 | *Sphrageidus similis* | 47 | 47 | 37 | 37 | 46 | 32 | 36 | 19 |
| K-48 | *Parasa sinica* | 3731 | 3732 | 2315 | 2315 | 3712 | 2243 | 3105 | 1538 |
| K-49 | *Pterodecta felderi* | 3701 | 3701 | 10865 | 10865 | 3673 | 10530 | 3011 | 8868 |
| K-50 | *Sibataniozephyrus fujisanus* | 1457 | 1457 | 520 | 520 | 1451 | 495 | 1124 | 307 |
| K-51 | *Colias erate* | 7063 | 7063 | 1787 | 1787 | 7024 | 1715 | 6181 | 1115 |
| K-52 | *Saturnia japonica* | 5343 | 5343 | 10599 | 10599 | 5322 | 10481 | 4500 | 8871 |
| K-53 | *Pterostoma gigantina* | 1410 | 1410 | 125 | 125 | 1396 | 115 | 1110 | 65 |
| K-54 | *Hypsomadius insignis* | 1097 | 1103 | 1986 | 1986 | 1095 | 1973 | 858 | 1338 |
| K-55 | *Papilio xuthus* | 1360 | 1360 | 2426 | 2426 | 1351 | 2395 | 1019 | 1520 |
| K-56 | *Kaniska canace* | 2517 | 2517 | 1588 | 1588 | 2502 | 1576 | 2009 | 1002 |
| K-57 | *Agrius convolvuli* | 6998 | 6998 | 15189 | 15189 | 6968 | 14477 | 5921 | 10323 |
| K-58 | *Terpnosia nigricosta* | 20930 | 20931 | 26482 | 26482 | 20842 | 25979 | 17050 | 18002 |
| K-59 | *Graptopsaltria nigrofuscata* | 9524 | 9526 | 13611 | 13611 | 9472 | 13421 | 6556 | 7179 |
| K-60 | *Platypleura kaempferi* | 5481 | 5481 | 6992 | 6992 | 5458 | 6901 | 3304 | 3833 |
| K-61 | *Sastragala esakii* | 197 | 197 | 87 | 87 | 196 | 82 | 137 | 33 |
| K-62 | *Leptoglossus occidentalis* | 567 | 568 | 147 | 147 | 561 | 145 | 404 | 74 |
| K-63 | *Palomena angulosa* | 2 | 2 | 4 | 4 | 2 | 3 | － | － |
| K-64 | *Ectrychotes andreae* | 90 | 90 | 7 | 7 | 90 | 6 | 65 | － |
| K-65 | *Bothrogonia ferruginea* | 234 | 234 | 84 | 84 | 232 | 68 | 150 | 26 |
| K-66 | *Agriosphodrus dohrni* | 26 | 26 | 150 | 150 | 26 | 129 | － | － |
| K-67 | *Notonecta triguttata* | 860 | 860 | 697 | 697 | 857 | 689 | 621 | 416 |
| K-68 | *Velinus nodipes* | － | － | 5 | 5 | － | 3 | － | － |
| K-69 | *Hesperocorixa hokkensis* | 4 | 4 | 2 | 2 | 4 | 1 | － | － |
| K-70 | *Pentatoma japonica* | 3 | 3 | 16 | 16 | 3 | 16 | － | 8 |
| K-71 | *Appasus major* | 123 | 123 | 171 | 171 | 123 | 163 | 88 | 93 |
| K-72 | *Appasus japonicus* | 299 | 299 | 1060 | 1060 | 297 | 1034 | 201 | 641 |
| K-73 | *Aquarius paludum* | 3 | 3 | 16 | 16 | 3 | － | － | － |
| K-74 | *Gryllotalpa orientalis* | 3552 | 3553 | 4882 | 4882 | 3533 | 4792 | 2842 | 3479 |
| K-75 | *Acrida cinerea* | 6465 | 6465 | 13185 | 13185 | 6433 | 12914 | 5446 | 9947 |
| K-76 | *Ruspolia dubia* | 418 | 418 | 557 | 557 | 418 | 547 | 271 | 317 |
| K-77 | *Oedaleus infernalis* | 1580 | 1580 | 851 | 851 | 1573 | 832 | 1095 | 441 |
| K-78 | *Locusta migratoria* | 4078 | 4078 | 1001 | 1001 | 4056 | 969 | 2739 | 452 |
| K-79 | *Aiolopus thalassinus* | 15771 | 15773 | 10046 | 10046 | 15694 | 9678 | 13476 | 7387 |
| K-80 | *Promachus yesonicus* | 3705 | 3705 | 2862 | 2862 | 3684 | 2817 | 3003 | 2058 |
| K-81 | *Neoitamus angusticornis* | 63 | 63 | 332 | 332 | 63 | 310 | － | － |
| K-82 | *Neoitamus sp.* | 19 | 19 | 16 | 16 | 19 | 9 | 16 | 5 |
| K-83 | *Tabanus chrysurus* | 219 | 219 | 175 | 175 | 219 | 162 | 144 | 79 |
| K-84 | *Sympetrum infuscatum* | 388 | 388 | 1187 | 1187 | 379 | 1150 | 268 | 712 |
| K-85 | *Sympetrum kunckeli* | 604 | 604 | 1971 | 1971 | 602 | 1949 | 449 | 1340 |
| K-86 | *Sympetrum darwinianum* | 127 | 127 | 187 | 187 | 126 | 175 | 87 | 107 |
| K-87 | *Davidius nanus* | 1327 | 1327 | 1689 | 1689 | 1322 | 1673 | 1009 | 1060 |
| K-88 | *Rhyothemis fuliginosa* | 2575 | 2578 | 3917 | 3917 | 2554 | 3857 | 2078 | 2880 |
| K-89 | *Paracercion hieroglyphicum* | 1174 | 1174 | 1490 | 1490 | 1172 | 1447 | 871 | 928 |
| K-90 | *Lestes sponsa* | 41 | 41 | 23 | 23 | 41 | 12 | 31 | 3 |
| K-91 | *Sympetrum frequens* | 1359 | 1359 | 2461 | 2461 | 1344 | 2422 | 999 | 1698 |
| K-92 | *Labidura riparia* | 3 | 3 | 153 | 153 | 3 | 146 | － | 39 |
| K-93 | *Myrmeleon bore* | 229 | 229 | 82 | 82 | 229 | 78 | 160 | 41 |
| K-94 | *Statilia maculata* | 1763 | 1764 | 299 | 299 | 1750 | 286 | 1328 | 156 |
| K-95 | *Tenodera aridifolia* | 61 | 61 | 1 | 1 | 61 | 1 | 46 | － |
| K-96 | *Periplaneta japonica* | 613 | 613 | 1088 | 1088 | 609 | 1073 | 395 | 631 |

^*1^The number of reads after the Claident command of *clsplitseq*. F and R represent forward and reverse side of the sequences, respectively.

^*2^The number of reads after the quality control by the Claident commend of *clfilterseq.*

^*3^The number of reads which were counted by the Claident command of *clsumclass*.
