# Supplementary material for: Development of mitochondrial DNA cytochrome c oxidase subunit I primer sets to construct DNA barcoding library using next-generation sequencing
Source: Biodivers Data J. 2024 Jun 18;12:e117014. doi: 10.3897/BDJ.12.e117014 (PMC11199957; doi:10.3897/BDJ.12.e117014)
Supplement: Supplementary material 3 — Phylogenetic tree using the neighbour-joining method [file bdj-12-e117014-s003.docx]

**Figure S1** The neighbour-joining (NJ) tree based of mtDNA COI region which was originated from sequence reads of the modified primer sets. The horizontal scale bar under the tree represents evolutionary distance between specimen taxa.
