# Supplementary material for: Development of mitochondrial DNA cytochrome c oxidase subunit I primer sets to construct DNA barcoding library using next-generation sequencing
Source: Biodivers Data J. 2024 Jun 18;12:e117014. doi: 10.3897/BDJ.12.e117014 (PMC11199957; doi:10.3897/BDJ.12.e117014)
Supplement: Supplementary material 4 — The number of reads from first half (LCO1490–COmfd_R: 1-319) and second half (COmfd_F–HCO2198: 262-658) of the COI region, and the results of BLAST search. [file bdj-12-e117014-s004.docx]

**Table S4** The number of reads from first half (LCO1490fd_F–COmfd_R: 1–319) and second half (COmfd_F–HCO2198rd_R: 262–658) of the COI region. In addition, the results of BLAST search.

|  | 1–319 | | 261–658 | | BLAST | |
| --- | --- | --- | --- | --- | --- | --- |
| species | 1st^*1^ | 2nd^*2^ | 1st^*1^ | 2nd^*2^ | species | Accession No. |
| *Carabus vanvolxemi* | 2839 | 15 | 3520 | 25 | *Carabus kumagaii* | AB047569.1 |
| *Carabus insulicola* | 4035 | 49 | 2954 | 14 | *Carabus albrechti hidakanus* | AB095195.1 |
| *Craspedonotus tibialis* | 149 | 1 | 102 | 1 | *Trichiorhyssemus aspergillus* | KU188370.1 |
| *Cylindera ovipennis* | 131 | 0 | 215 | 1 | *Cylindera arenaria* | KM442873.1 |
| *Scarites terricola* | 6141 | 2265 | 2384 | 1861 | *Scarites aterrimus* | JF713795.1 |
| *Pterostichus sp.* | 3746 | 0 | 1547 | 23 | *Pterostichus agonus* | KU876029.1 |
| *Acalolepta luxuriosa* | 130 | 6 | 24 | 5 | *Acalolepta sejuncta* | LC617387.1 |
| *Anoplophora chinensis* | 18024 | 534 | 15712 | 565 | *Anoplophora chinensis* | AB439166.1 |
| *Batocera lineolata* | 7707 | 128 | 2946 | 1109 | *Batocera lineolata* | KY357614.1 |
| *Prionus insularis* | 980 | 0 | 610 | 0 | *Prionus insularis* | LC617379.1 |
| *Aiolocaria hexaspilota* | 23 | 0 | 24 | 0 | *Aiolocaria hexaspilota* | OL664076.1 |
| *Cybister chinensis* | 109 | 0 | 2 | 0 | *Cybister chinensis* | LC727364.1 |
| *Graphoderus adamsii* | 57 | 0 | 9 | 0 | *Graphoderus adamsii* | LC727333.1 |
| *Rhantus suturalis* | － | － | － | － | － | － |
| *Hydrochara affinis* | 8020 | 1 | 7971 | 0 | *Hydrochara affinis* | LC797102.1 |
| *Hydrophilus acuminatus* | 8335 | 103 | 11208 | 62 | *Hydrophilus acuminatus* | LC659994.1 |
| *Dorcus rectus* | 326 | 150 | 835 | 38 | *Dorcus rectus* | LC619083.1 |
| *Prosopocoilus inclinatus* | 1184 | 1 | 2856 | 1 | *Prosopocoilus inclinatus* | MN609395.1 |
| *Sipalinus gigas* | 2783 | 30 | 1162 | 9 | *Sipalinus gigas* | LC492874.1 |
| *Anomala schoenfeldti* | 536 | 5 | 337 | 2 | *Anomala rufocuprea* | LC619110.1 |
| *Polyphylla albolineata* | 1525 | 84 | 3223 | 149 | *Polyphylla gracilicornis* | NC_054285.1 |
| *Protaetia orientalis* | 5218 | 0 | 7267 | 7007 | *Protaetia orientalis* | MW085777.1 |
| *Trypoxylus dichotomus* | 1673 | 1 | 1417 | 11 | *Trypoxylus dichotomus* | OL343388.1 |
| *Cryphaeus amurensis* | － | － | － | － | － | － |
| *Apis mellifera* | 66 | 31 | 1727 | 0 | *Apis mellifera* | OP890239.1 |
| *Bombus diversus* | 608 | 25 | 727 | 0 | *Bombus diversus* | HQ553053.1 |
| *Bombus terrestris* | － | － | － | － | － | － |
| *Auplopus carbonarius* | － | － | － | － | － | － |
| *Scolia histrionica* | － | － | － | － | － | － |
| *Oreumenes decoratus* | 443 | 3 | 268 | 4 | *Oreumenes decoratus* | OL343142.1 |
| *Parapolybia indica* | 1612 | 2 | 1106 | 3 | *Parapolybia crocea* | NC_036343.1 |
| *Polistes chinensis antennalis* | 26 | 1 | 9 | 0 | *Polistes chinensis antennalis* | AB795286.1 |
| *Polistes jokahamae* | 3867 | 153 | 3411 | 63 | *Polistes jokahamae* | AB969806.1 |
| *Polistes rothneyi* | 626 | 0 | 338 | 5 | *Polistes rothneyi* | OM991970.1 |
| *Vespa analis* | 1090 | 775 | 868 | 387 | *Vespa analis* | LC728285.1 |
| *Vespa ducalis* | 1572 | 0 | 1669 | 0 | *Vespa ducalis* | MN716818.1 |
| *Vespa mandarinia* | 7871 | 11 | 12385 | 14 | *Vespa mandarinia* | AB851894.1 |
| *Sarbanissa subflava* | 4388 | 0 | 2931 | 1 | *Sarbanissa subflava* | JN087392.1 |
| *Pterodecta felderi* | 3011 | 4 | 8868 | 25 | *Pterodecta felderi* | KF492061.1 |
| *Hypsomadius insignis* | 858 | 0 | 1338 | 0 | *Hypsomadius insignis* | FJ768745.1 |
| *Parasa sinica* | 3105 | 0 | 1538 | 1 | *Parasa sinica* | KJ638149.1 |
| *Lycaena phlaeas* | 48 | 2 | － | － | － | － |
| *Hypaurotis fujisanus* | 1124 | 0 | 307 | 0 | *Hypaurotis fujisanus* | ON436068.1 |
| *Euproctis similis* | 36 | 0 | 19 | 0 | *Euproctis similis* | HM872108.1 |
| *Pterostoma gigantina* | 1110 | 0 | 65 | 0 | *Trichodezia albovittata* | HM415494.1 |
| *Nymphalis canace* | 2009 | 0 | 1002 | 14 | *Nymphalis canace* | NC_058606.1 |
| *Minois dryas* | 1000 | 0 | 503 | 0 | *Minois dryas* | EF545703.1 |
| *Neope niphonica* | 10303 | 14 | 8532 | 45 | *Neope bremeri* | DQ338770.1 |
| *Ochlodes ochracea* | 1295 | 0 | 2339 | 0 | *Ochlodes ochracea* | OQ749884.1 |
| *Vanessa indica* | 12816 | 149 | 5480 | 125 | *Vanessa indica* | OK342128.1 |
| *Papilio xuthus* | 1019 | 0 | 1520 | 67 | *Papilio xuthus* | GU372543.1 |
| *Parnassius citrinarius* | 8379 | 25 | 11203 | 24 | *Parnassius glacialis* | AM231428.1 |
| *Colias erate* | 6181 | 8 | 1115 | 15 | *Colias erate* | GU372561.1 |
| *Pieris melete* | 4541 | 0 | 6119 | 0 | *Pieris melete* | OL664160.1 |
| *Saturnia japonica* | 4500 | 0 | 8871 | 0 | *Saturnia japonica* | OL664256.1 |
| *Agrius convolvuli* | 5921 | 0 | 10323 | 1273 | *Agrius convolvuli* | LC049959.1 |
| *Smerinthus planus* | 4596 | 0 | 703 | 259 | *Smerinthus planus* | MW085706.1 |
| *Sastragala esakii* | 137 | 0 | 33 | 0 | *Sastragala esakii* | KC510112.1 |
| *Appasus japonicus* | 201 | 4 | 641 | 4 | *Appasus japonicus* | LC548505.1 |
| *Appasus major* | 88 | 5 | 93 | 6 | *Appasus major* | AB742728.1 |
| *Bothrogonia ferruginea* | 150 | 0 | 26 | 1 | *Bothrogonia ferruginea* | KU167550.1 |
| *Graptopsaltria nigrofuscata* | 6556 | 408 | 7179 | 502 | *Graptopsaltria nigrofuscata* | AB900647.1 |
| *Platypleura kaempferi* | 3304 | 284 | 3833 | 270 | *Platypleura kaempferi* | OQ825986.1 |
| *Yezoterpnosia nigricosta* | 17050 | 223 | 18002 | 707 | *Yezoterpnosia nigricosta* | AB900645.1 |
| *Leptoglossus occidentalis* | 404 | 0 | 74 | 0 | *Leptoglossus occidentalis* | HQ105829.1 |
| *Hesperocorixa hokkensis* | － | － | － | － | － | － |
| *Aquarius paludum* | － | － | － | － | － | － |
| *Notonecta triguttata* | 621 | 1 | 416 | 1 | *Notonecta triguttata* | NC_070247.1 |
| *Palomena angulosa* | － | － | － | － | － | － |
| *Pentatoma japonica* | － | － | 8 | 0 | － | － |
| *Agriosphodrus dohrni* | － | － | － | － | － | － |
| *Ectrychotes andreae* | 65 | 0 | － | － | － | － |
| *Velinus nodipes* | － | － | － | － | － | － |
| *Acrida cinerea* | 5446 | 11 | 9947 | 145 | *Acrida cinerea* | OQ213928.1 |
| *Aiolopus thalassinus* | 13476 | 33 | 7387 | 40 | *Aiolopus thalassinus* | KC140014.1 |
| *Locusta migratoria* | 2739 | 24 | 452 | 24 | *Locusta migratoria* | JN858297.1 |
| *Oedaleus infernalis* | 1095 | 6 | 441 | 2 | *Oedaleus infernalis* | OQ214124.1 |
| *Gryllotalpa orientalis* | 2842 | 11 | 3479 | 20 | *Gryllotalpa orientalis* | OP271488.1 |
| *Ruspolia dubia* | 271 | 1 | 317 | 0 | *Ruspolia dubia* | JQ793673.1 |
| *Neoitamus angusticornis* | － | － | － | － | － | － |
| *Neoitamus sp.* | 16 | 0 | 5 | 0 | *Neoitamus angusticornis* | OL343358.1 |
| *Promachus yesonicus* | 3003 | 0 | 2058 | 8 | *Promachus albifacies* | KT733437.1 |
| *Tabanus chrysurus* | 144 | 1 | 79 | 0 | *Tabanus chrysurus* | MW085770.1 |
| *Paracercion hieroglyphicum* | 871 | 0 | 928 | 5 | *Paracercion hieroglyphicum* | MW361622.1 |
| *Davidius nanus* | 1009 | 0 | 1060 | 3 | *Davidius nanus* | MK774338.1 |
| *Lestes sponsa* | 31 | 0 | 3 | 1 | *Lestes sponsa* | MW490394.1 |
| *Rhyothemis fuliginosa* | 2078 | 0 | 2880 | 1 | *Rhyothemis fuliginosa* | AB709109.1 |
| *Sympetrum darwinianum* | 87 | 0 | 107 | 0 | *Sympetrum darwinianum* | MK774310.1 |
| *Sympetrum frequens* | 999 | 20 | 1698 | 3 | *Sympetrum frequens* | KF257076.1 |
| *Sympetrum infuscatum* | 268 | 0 | 712 | 7 | *Sympetrum infuscatum* | KF257078.1 |
| *Sympetrum kunckeli* | 449 | 0 | 1340 | 0 | *Sympetrum kunckeli* | KF257092.1 |
| *Labidura riparia* | － | － | 39 | 29 | － | － |
| *Myrmeleon bore* | 160 | 0 | 41 | 0 | *Myrmeleon bore* | KJ592439.1 |
| Statilia maculata | 1328 | 4 | 156 | 0 | *Statilia maculata* | MW085640.1 |
| Tenodera aridifolia | 46 | 0 | － | － | － | － |
| Periplaneta japonica | 395 | 27 | 631 | 6 | *Periplaneta japonica* | JQ350708.1 |

^*1^The number of reads of the most dominant in the total reads.

^*2^The number of reads of the second largest in the total reads.
