# Supplementary material for: Development of mitochondrial DNA cytochrome c oxidase subunit I primer sets to construct DNA barcoding library using next-generation sequencing
Source: Biodivers Data J. 2024 Jun 18;12:e117014. doi: 10.3897/BDJ.12.e117014 (PMC11199957; doi:10.3897/BDJ.12.e117014)
Supplement: Supplementary material 5 — Comparison results of Chironomid NGS assemblies against the sequence data from the Chironomid DNA Barcode Database by the National Institute for Environmental Studies (NIES). [file bdj-12-e117014-s005.docx]

**Table S5** Comparison results of Chironomid NGS assemblies against the sequence data from the Chironomid DNA Barcode Database by the National Institute for Environmental Studies (NIES).

| Sample ID | Species | NGS sequencing^*1^ | Accession No. on DDBJ | Specimen ID^*2^ | Identities^*3^ |
| --- | --- | --- | --- | --- | --- |
| D0002 | *Chironomus plumosus* | + | AB740253 | D0002 | 658/658 (100%) |
| D0004 | *Lipiniella moderata* | + | LC096199 | D0004 | 658/658 (100%) |
| D0006 | *Cricotopus sylvestris* | + | AB838601 | D0006 | 658/658 (100%) |
| D0007 | *Cricotopus sylvestris* | + | AB838605 | D0007 | 658/658 (100%) |
| D0008 | *Cricotopus sylvestris* | + | AB838601 | D0008 | 658/658 (100%) |
| D0010 | *Dicrotendipes pelochloris* | + | AB740264 | D0010 | 658/658 (100%) |
| D0012 | *Dicrotendipes pelochloris* | + | AB838653 | D0012 | 658/658 (100%) |
| D0014 | *Tanypus nakazatoi* | + | AB838639 | D0014 | 658/658 (100%) |
| D0015 | *Tanypus nakazatoi* | + | AB838640 | D0015 | 658/658 (100%) |
| D0016 | *Chironomus plumosus* | – | AB740253 | D0016 | – |
| D0018 | *Lipiniella moderata* | + | AB838668 | D0018 | 658/658 (100%) |
| D0020 | *Propsilocerus akamusi* | + | AB838625 | D0020 | 658/658 (100%) |
| D0022 | *Chironomus plumosus* | – | AB740253 | D0022 | – |
| D0060 | *Chironomus nippodorsalis* | + | AB740242 | D0060 | 658/658 (100%) |
| D0061 | *Chironomus nippodorsalis* | – | AB740242 | D0061 | – |
| D0162 | *Propsilocerus akamusi* | + | AB838625 | D0162 | 658/658 (100%) |

^*1^ Results of NGS sequencing: “+” represents presence of sequence reads and “–” represents absence of sequence reads.

^*2^ The specimen ID of the database.

^*3^ Identity rate between the NGS assembly and the sequence data from the database. The unit of the numbers are base pair (bp).
